# Perioperative and Mid-Term Outcomes of Primary Versus Redo Pericardiectomy for Constrictive Pericarditis: A Single-Center Retrospective Cohort Study

**DOI:** 10.3390/jcm15187223

**Published:** 2026-09-17

**Authors:** Da Gong, Can Zhao, Chao Dong, Jianping Xu, Jin Wang, Guangyu Pan

**Affiliations:** 1 Department of Cardiac Surgery, Peking University First Hospital, Beijing 100035, China; gdletter@pku.org.cn; 2 Department of Cardiac Surgery, Peking University International Hospital, Beijing 102200, China; 3 Department of Cardiac Surgery, Peking University People’s Hospital, Beijing 100032, China

**Keywords:** constrictive pericarditis, pericardiectomy, redo pericardiectomy, low cardiac output syndrome, acute kidney injury, prognosis

## Abstract

**Background**: Direct comparative evidence between redo and primary pericardiectomy for constrictive pericarditis (CP) is limited to small, uncontrolled series. We compared perioperative and mid-term outcomes between the two cohorts and explored factors associated with all-cause death. **Methods**: In this single-center retrospective cohort study, 137 consecutive patients with CP undergoing radical pericardiectomy between April 2018 and December 2023 were allocated to a primary pericardiectomy cohort (PPC, *n* = 86) or a redo pericardiectomy cohort (RPC, *n* = 51). The primary endpoint was all-cause death; secondary endpoints included low cardiac output syndrome (LCOS), unplanned reoperation, acute kidney injury (AKI), atrial fibrillation, and postoperative New York Heart Association class. Survival was analyzed with Kaplan–Meier curves and log-rank tests, prognostic factors with Firth-penalized Cox regression (with baseline adjustment and multiple imputation), and the origin of the survival difference was explored via a sensitivity analysis excluding operative deaths. **Results**: The RPC had more preoperative atrial fibrillation (64.7% vs. 36.0%, *p* = 0.002) and a larger left atrial diameter (54.8 vs. 47.0 mm, *p* < 0.001) than the PPC. Operative mortality was higher in the RPC (15.7% vs. 2.3%; relative risk 6.745, *p* = 0.006), as was LCOS (13.7% vs. 3.5%, *p* = 0.026). Seventeen patients died (6 PPC, 11 RPC) over a median follow-up of 3.6 years; cumulative overall survival was lower in the RPC (log-rank *p* = 0.013; 3-year survival 78.4% vs. 94.2%). Postoperative LCOS was the strongest independent predictor of death (hazard ratio [HR] 22.526, *p* < 0.001); redo pericardiectomy (HR 3.031; 95% CI 1.034–9.679; *p* = 0.043) and AKI stage (HR 1.848; 95% CI 1.107–3.088; *p* = 0.020) were independently associated with death; postoperative albumin showed a borderline protective association (HR 0.913, *p* = 0.048). Among perioperative survivors, no statistically significant difference in long-term survival was detected (HR 1.275; 95% CI 0.279–5.819; *p* = 0.753). **Conclusions**: Redo pericardiectomy was associated with substantially higher perioperative mortality; among perioperative survivors, long-term survival did not differ detectably between cohorts, although estimates were imprecise. Because the excess risk was accompanied by potentially modifiable perioperative events, intensified risk assessment and meticulous perioperative management warrant evaluation as approaches to improving outcomes in this high-risk population.

## 1. Introduction

Constrictive pericarditis (CP) is an end-stage pericardial disease characterized by fibrosis and calcification of the pericardium with resultant restriction of ventricular diastole; the loss of pericardial compliance impedes ventricular filling and produces a syndrome of systemic venous congestion dominated by right-sided heart failure [1,2]. The etiological spectrum of CP varies considerably across regions and eras: tuberculous pericarditis remains the leading cause in developing countries, whereas post-surgical and post-radiation constriction are increasingly prevalent in developed countries, with idiopathic CP also accounting for a substantial proportion [1]. Without surgical intervention, the natural history of CP is bleak, and most patients eventually die of chronic heart failure and its complications [3].

Pericardiectomy is currently the only potentially curative treatment; its central objective is to relieve the restraint on the cardiac diastole and restore normal hemodynamics through complete excision of the diseased pericardium. Radical pericardiectomy is defined as removal of the entire pericardium except for the narrow strip of pericardium along the course of the phrenic nerves, or excision of that strip after the phrenic nerve has been dissected free [4]. Contemporary series report perioperative mortality of 4–11% after primary pericardiectomy, with long-term survival exceeding 80% [5,6,7]. Nevertheless, even after a technically successful primary operation, some patients develop recurrent symptoms attributable to residual pericardial constriction, progression of epicardial fibrosis, persistent postoperative inflammation, or an inadequate extent of the initial decortication, and further surgical intervention must be considered [8,9].

Redo pericardiectomy generally refers to repeat surgical decortication in patients who have previously undergone pericardiectomy, undertaken for residual or recurrent constriction, inadequate localized decortication, progressive postoperative inflammation, or aggravation of epicardial or myocardial fibrosis [9]. Compared with the primary operation, redo surgery is frequently accompanied by dense intrathoracic and mediastinal adhesions, distorted anatomy, and increased risks of myocardial or coronary injury, and its technical difficulty and perioperative risk are substantially higher. However, available studies on redo pericardiectomy are largely small series of 10–40 patients, and direct comparison with contemporaneous primary operations is lacking [9,10,11]. In the absence of controlled data, surgeons facing the decision to reoperate can rely only on empirical judgment, and an objective risk-benefit assessment for the individual patient is difficult.

We therefore assembled what is, to our knowledge, the largest reported cohort of patients undergoing redo pericardiectomy and directly compared short- and mid-term clinical outcomes between primary and redo pericardiectomy performed at a single center over the same period. By combining multivariable Cox regression with a prespecified sensitivity analysis, we sought to identify the independent determinants of prognosis and to localize the source of any survival difference, thereby providing more reliable evidence for surgical decision-making, perioperative management, and prognostic evaluation in this high-risk population.

## 2. Patients and Methods

### 2.1. Study Design and Population

This single-center retrospective cohort study consecutively enrolled patients diagnosed with CP who underwent radical pericardiectomy in the Department of Cardiac Surgery, Peking University International Hospital, between April 2018 and December 2023. Taking the type of surgery as the exposure, patients were allocated to a primary pericardiectomy cohort (PPC) or a redo pericardiectomy cohort (RPC), and perioperative outcomes and mid-term prognosis were compared between the two cohorts. The study is reported in accordance with the STROBE recommendations for observational studies.

The inclusion criteria were: (1) age ≥ 18 years; (2) CP confirmed by clinical presentation and imaging; and (3) radical pericardiectomy performed at our center during the study period, with concomitant valve replacement or repair, coronary artery bypass grafting, or other cardiac procedures permitted. The exclusion criteria were: (1) restrictive cardiomyopathy or any other disease that adequately explains restricted ventricular diastole without a predominantly pericardial etiology; (2) previous palliative interventions only, such as pericardial window, pericardiocentesis with drainage, or limited pericardial release, without complete pericardiectomy; (3) severe missing perioperative data precluding accurate assessment of perioperative outcomes; and (4) loss to follow-up or severely incomplete follow-up data precluding assessment of mid-term prognosis. Exclusion criteria (2), concerning previous palliative interventions without a complete pericardiectomy (pericardial window, pericardiocentesis with drainage, or limited pericardial release), was prespecified; however, no patient screened during the study period was excluded under this criterion. All redo patients in the series had therefore previously undergone a formal pericardiectomy, and the redo procedures in this cohort largely represent completion procedures for residual or recurrent constriction.

The diagnosis of CP was based on clinical features of right heart failure together with echocardiographic findings of pericardial thickening and constrictive physiology (septal bounce, respiratory variation in ventricular filling, and dilated inferior vena cava). Chest CT was obtained to document pericardial thickening or calcification when clinically indicated. The diagnosis was confirmed intraoperatively in all patients. Cardiac MRI and invasive hemodynamic assessment (right heart catheterization) were reserved for selected cases in which the diagnosis was uncertain, or a restrictive cardiomyopathy had to be excluded.

Patients were assigned to the redo pericardiectomy cohort (RPC) if they had previously undergone pericardiectomy and were undergoing repeat radical pericardiectomy for constrictive disease. All redo patients in this series had undergone initial pericardiectomy at outside institutions a median of 15 years before the redo procedure (IQR 9.5–29.0, range 0.3–43 years). Because these operations predated the establishment of the present centre, operative records of the initial procedures could not be fully retrieved, and the precise extent of the initial resection could not be verified. The initial approach was a median sternotomy in 49 patients and a left anterolateral thoracotomy in 2. The indication for redo surgery was residual or recurrent constriction: recurrence or persistence of right-heart failure symptoms (peripheral edema, ascites, and reduced exercise tolerance), together with echocardiographic and CT evidence of persistent or recurrent constriction, confirmed intraoperatively. Because operative records of the initial procedure were unavailable, residual and recurrent constriction could not be reliably distinguished; the indication for redo surgery was therefore defined by the combination of recurrent symptoms, imaging evidence, and intraoperative confirmation of constriction.

All diagnoses of constrictive pericarditis were confirmed by histology of the resected pericardium at operation. The cause of CP was then assigned on the basis of each patient’s clinical history, supported by the available pathology. A patient with a documented history of tuberculosis was classified as having tuberculous CP, which was the largest single cause in this cohort (51.8%). Radiation-induced CP was assigned when there was a documented history of thoracic radiotherapy. Pericardial malignancy was confirmed on histology of the resected pericardium; the single pericardial mesothelioma was diagnosed in this way. Patients without an identifiable cause after this assessment were classified as having idiopathic CP.

### 2.2. Surgical Technique

All patients underwent radical pericardiectomy as defined above. Cardiopulmonary bypass (CPB) was used at the discretion of the operating surgeon, and concomitant valve or coronary artery bypass grafting procedures were performed when indicated.

All operations were performed through a median sternotomy, and all were elective, by one of three senior surgeons (C.D., J.X., G.P.) of the same departmental team. Radical pericardiectomy was defined as resection of the pericardium between the two phrenic nerves, leaving a narrow strip (~1 cm) anterior to each phrenic nerve, extending cephalad to the pericardial reflections and caudally across the entire diaphragmatic surface; constricting bands around the superior and inferior venae cavae were divided. The mediastinal pleurae were opened bilaterally when necessary to drain pleural effusions and to reduce the likelihood of later re-constriction. Because the visceral and parietal layers are fused in CP, decortication was carried down to the epicardial fat layer or until the underlying myocardium became faintly visible; dissection into the epicardium itself was avoided. The procedure was judged complete when both ventricles, the right atrium, and both caval orifices were fully released.

Transesophageal echocardiography (TEE) was used in all procedures to monitor cardiac function, volume status, and valve function in real time; the decision to perform a concomitant valve procedure was made on the basis of TEE findings together with the preoperative echocardiogram. Cardiopulmonary bypass (CPB) was used for all procedures with a concomitant valve or coronary procedure and was available during isolated pericardiectomy, being instituted at the surgeon’s discretion when dissection was difficult or hemodynamics became unstable.

### 2.3. Data Collection

Clinical data were collected through the electronic medical record system in four categories. Baseline data included age, sex, height, weight, body mass index (BMI), and preoperative New York Heart Association (NYHA) class. Laboratory indices included hemoglobin, albumin, creatinine, blood urea nitrogen, uric acid, and estimated glomerular filtration rate (eGFR). eGFR was computed using the Chronic Kidney Disease Epidemiology Collaboration (CKD-EPI) 2009 creatinine equation as reported by the laboratory information system. Ancillary examinations included left ventricular ejection fraction (LVEF), left atrial diameter (LAD), left ventricular end-diastolic diameter (LVEDD), and electrocardiography. Operative data included American Society of Anesthesiologists (ASA) class, procedure type, operative time, central venous pressure (CVP), and CPB-related parameters. Preoperative laboratory values were the last values obtained before surgery during the index admission. Postoperative laboratory values were measured on the first postoperative morning (the first morning blood draw after ICU admission, taken between 06:00 and 07:00).

### 2.4. Definitions

Low cardiac output syndrome (LCOS) denotes a clinical syndrome of decreased cardiac output with hypoperfusion of peripheral organs, typically manifested as hypotension, tachycardia, oliguria, metabolic acidosis with elevated blood lactate, and pale, moist skin with cold extremities [12]. Operationally, LCOS was adjudicated when a postoperative low-output state required continuous inotropic support or mechanical circulatory support with an intra-aortic balloon pump or extracorporeal membrane oxygenation to maintain circulatory stability. LCOS was adjudicated during the early postoperative ICU course.

Unplanned reoperation was defined as any reintervention during the index hospitalization that was undertaken for a surgery-related complication and had not been planned preoperatively, mainly including reexploration for bleeding and redo valve surgery; pre-planned staged operations were not counted.

Acute kidney injury (AKI) was diagnosed and staged according to the 2012 Kidney Disease: Improving Global Outcomes (KDIGO) guideline: an increase in serum creatinine of ≥26.5 μmol/L within 48 h, an increase to ≥1.5 times baseline within 7 days, or urine output <0.5 mL/kg/hour for ≥6 h. AKI was further staged 1–3 according to the magnitude of creatinine elevation and oliguria, with higher stages indicating more severe injury. AKI was adjudicated within the first 7 postoperative days.

Operative mortality was defined according to the composite endpoint of the American College of Surgeons National Surgical Quality Improvement Program (ACS NSQIP) operations manual: death from any cause within 30 days after surgery, regardless of discharge status. If the postoperative hospitalization exceeded 30 days, all deaths during that hospitalization were included. The definition uses all-cause mortality without cause-of-death adjudication to minimize ascertainment bias. Operative mortality and postoperative complications were extracted by two investigators independently from the medical records in accordance with the prespecified definitions (operative mortality by the ACS NSQIP definition; AKI by KDIGO criteria; LCOS by the requirement for continuous inotropic or mechanical circulatory support). Discrepancies were resolved by joint review. Because this is a retrospective study, formal independent endpoint adjudication was not performed.

### 2.5. Follow-Up and Endpoints

**Figure 1:**
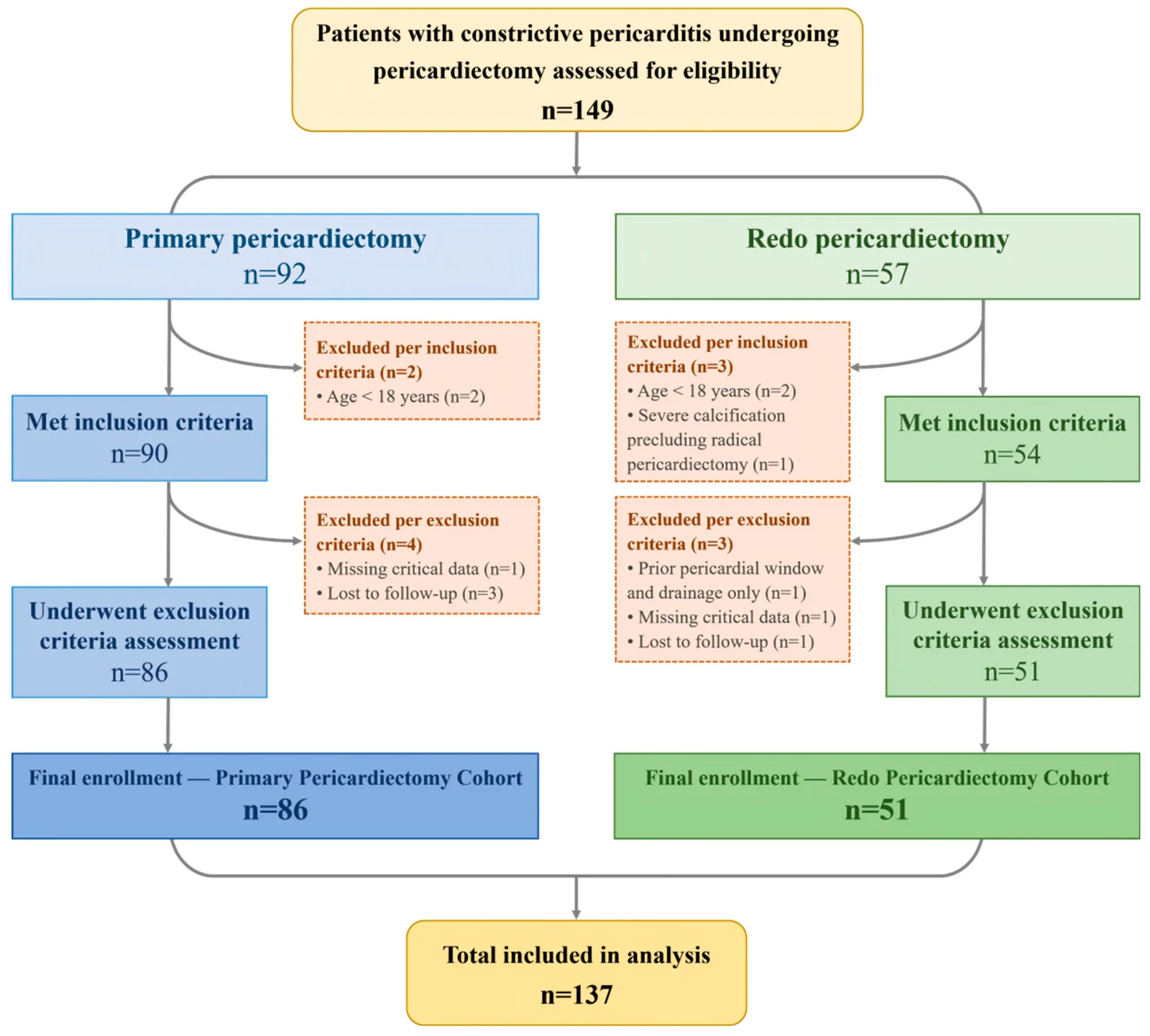
Flow diagram of patient enrollment. Of 149 consecutive patients with constrictive pericarditis undergoing pericardiectomy between April 2018 and December 2023, 12 were excluded (5 for inclusion criteria; 7 for exclusion criteria). Four of these exclusions were for complete loss to follow-up. Three were PPC patients (one last contacted at 1 year; two at 6 months after surgery) and one was a prospective RPC patient (last contacted at 1 year). All four lived in remote areas of China and were unreachable by telephone or clinic visit; their vital status after the last contact is unknown. The remaining analyses are based on 137 patients, 86 in the primary pericardiectomy cohort (PPC) and 51 in the redo pericardiectomy cohort (RPC).

Follow-up was performed through the electronic medical record system combined with telephone interviews, and survival status, symptoms, NYHA class, and medications were recorded. Because follow-up availability is itself a post-baseline selection step, patients whose vital status could not be ascertained at all were excluded from the primary analysis rather than censored; for complete transparency, their numbers, distribution by cohort, and last-known follow-up durations are reported in Section 3.1 (Figure 1), and the robustness of excluding them was tested in a sensitivity analysis.

The primary endpoint was all-cause death during follow-up. Secondary endpoints comprised perioperative and follow-up morbidity: (1) LCOS; (2) unplanned reoperation; (3) AKI; (4) atrial fibrillation; and (5) postoperative NYHA class. Atrial fibrillation (AF) and NYHA class were assessed at the last documented contact for each patient.

### 2.6. Statistical Analysis

Analyses were performed with IBM SPSS Statistics (version 29.0) and R (version 4.6.1; packages survival, coxphf, and mice); figures were prepared with GraphPad Prism (version 9.3.0). All continuous variables deviated from a normal distribution and are presented as the median (25th and 75th percentiles); between-group comparisons used the Mann–Whitney U test, and within-group comparisons used the Wilcoxon signed-rank test. Categorical variables are presented as *n* (%) and were compared with the chi-square test or Fisher’s exact test, as appropriate. Survival was estimated by the Kaplan–Meier method and compared with the log-rank test. The association between redo surgery and all-cause death was first estimated with a univariable Cox proportional hazards model.

#### 2.6.1. Multivariable Modeling

Candidate covariates comprised all 33 measured demographic, preoperative, intraoperative, and postoperative variables (Supplementary Table S1); the etiologic group was excluded because two rare subgroups were universally fatal. Selection was data-driven. Covariates associated with death at *p* < 0.10 on univariable Firth-penalized Cox screening entered multivariable modeling, followed by backward elimination and pruning of correlated measures of the same construct. Given the limited number of events, and because all 10 patients with low cardiac output syndrome (LCOS) died (quasi-complete separation with the outcome), multivariable analysis used Firth-penalized likelihood Cox regression to obtain finite estimates and reduce small-sample bias. The proportional-hazards assumption was examined with Schoenfeld residuals; linearity of continuous predictors was examined with restricted cubic splines; collinearity was assessed with variance inflation factors; and influential observations were assessed with dfbetas and drop-one analyses.

#### 2.6.2. Missing Data

Eighteen candidate covariates had incomplete values (0.7–4.4% per variable), whereas the exposure, outcomes, and follow-up time were complete, and no patient was lost to follow-up after enrollment; variable-specific counts and proportions by cohort are given in Supplementary Table S2. Missing values were imputed by multiple imputation with chained equations (m = 20) using predictive mean matching. The imputation model included all candidate covariates, survival time, and the event indicator, and estimates were pooled across imputed datasets according to Rubin’s rules. Missingness was not associated with vital status or cohort allocation for any incomplete variable (Fisher exact tests, all *p* > 0.10), consistent with the missing-at-random (MAR) assumption.

#### 2.6.3. Model Performance and Internal Validation

Discrimination was assessed with the concordance index (C-index), and model fit with the Akaike information criterion (AIC). Internal validation was performed by bootstrap resampling (500 replications) to obtain the optimism-corrected C-index.

#### 2.6.4. Sensitivity Analysis

A sensitivity analysis excluding operative deaths was conducted to determine the extent to which early deaths accounted for the long-term survival difference. Landmark survival analyses beginning at day 30 and at hospital discharge were performed with effect estimates and 95% confidence intervals. All tests were two-sided, with α = 0.05.

### 2.7. Ethics

The study protocol was approved by the Biomedical Ethics Committee of Peking University International Hospital (approval No. 2024-KY-0001-01; date of approval: 22 February 2024), and the requirement for individual informed consent was waived. The study was conducted in accordance with the Declaration of Helsinki.

## 3. Results

### 3.1. Patient Enrollment and Baseline Characteristics

Of the 149 consecutive patients with CP who underwent pericardiectomy during the study period, 12 were excluded (5 not meeting the inclusion criteria and 7 meeting the exclusion criteria), leaving 137 patients for the final analysis: 86 in the PPC and 51 in the RPC (Figure 1). Baseline characteristics are summarized in Table 1. Sex distribution did not differ between cohorts. Patients in the RPC were significantly younger (median 43 vs. 53 years, *p* = 0.023) and had a lower BMI (*p* = 0.046); the median interval since the initial pericardiectomy was 15.0 (9.5–29.0) years. Preoperative NYHA class, preoperative hospital stay, diuresis-induced weight loss, LVEF, and LVEDD were comparable between cohorts. Laboratory profiles were similar except for a higher eGFR in the RPC (99.7 vs. 89.1 mL/min/1.73 m^2^, *p* = 0.004). The RPC had a markedly higher prevalence of preoperative atrial fibrillation (64.7% vs. 36.0%, *p* = 0.002) and, concordantly, a larger LAD (54.8 vs. 47.0 mm, *p* < 0.001).

### 3.2. Perioperative Data

Perioperative data are shown in Table 2. The distribution of procedure type (isolated pericardiectomy vs. concomitant procedures), use of CPB, ASA class, preoperative and postoperative CVP, postoperative and total hospital stay, postoperative LVEF and LVEDD, etiological composition, pre-discharge diuretic weight loss, and postoperative laboratory indices did not differ between cohorts. CVP decreased significantly after surgery in both cohorts (PPC, 15.0 to 5.5 cm H_2_O; RPC, 12.0 to 5.0 cm H_2_O; both *p* < 0.001), and LAD also decreased significantly in both (*p* < 0.05), confirming effective relief of constriction; nevertheless, postoperative LAD remained larger in the RPC (46.0 vs. 39.0 mm, *p* < 0.001). Subgroup analysis of operative time showed no difference for isolated pericardiectomy (210.0 vs. 200.0 min, *p* = 0.263), whereas among patients undergoing concomitant valve or coronary procedures, operative time was significantly longer in the RPC (382.5 vs. 330.0 min, *p* = 0.030). When the difference across these strata was examined formally by including a redo × procedure-type interaction term in a log-linear model of operative time, the interaction was not statistically significant (*p* = 0.551); therefore, the pattern of a greater primary difference in redo surgeries and complex scenarios should be regarded as exploratory rather than confirmatory.

Among on-pump procedures, CPB time (median [IQR] 147 (131–222) vs. 96 (79–122) min; *p* < 0.001) and aortic cross-clamp time (80 (69–102) vs. 62 (47–72) min; *p* < 0.001) were significantly longer in the RPC than in the PPC; assisted perfusion time was also longer in the RPC, but the difference did not reach statistical significance (39 (27–55) vs. 32 (20–38) min; *p* = 0.103).

Five notable perioperative events were recorded. One PPC patient required a second CPB run because of an unsatisfactory valve repair; the patient recovered and was alive with NYHA class II at last follow-up. Among RPC patients, one required two additional CPB runs for recurrent ventricular fibrillation and low cardiac output during dissection and died of low output on postoperative day 2; one sustained injury to the posterior aortic wall and died 3.8 days postoperatively; and two underwent isolated pericardiectomy with no valve abnormality on intraoperative TEE but developed valve dysfunction with hemodynamic deterioration in the ICU, requiring unplanned mitral and tricuspid valve repair on postoperative days 7 and 22, respectively. Both patients were discharged alive but died on postoperative days 99 and 296. Intraoperative TEE immediately after decortication had shown normal valve function in both patients; the delayed functional deterioration may reflect geometric rearrangement of the atrioventricular apparatus after pericardial release—altered annular dimensions and subvalvular tension as ventricular geometry expands—rather than direct valve injury during dissection.

### 3.3. Survival

**Figure 2:**
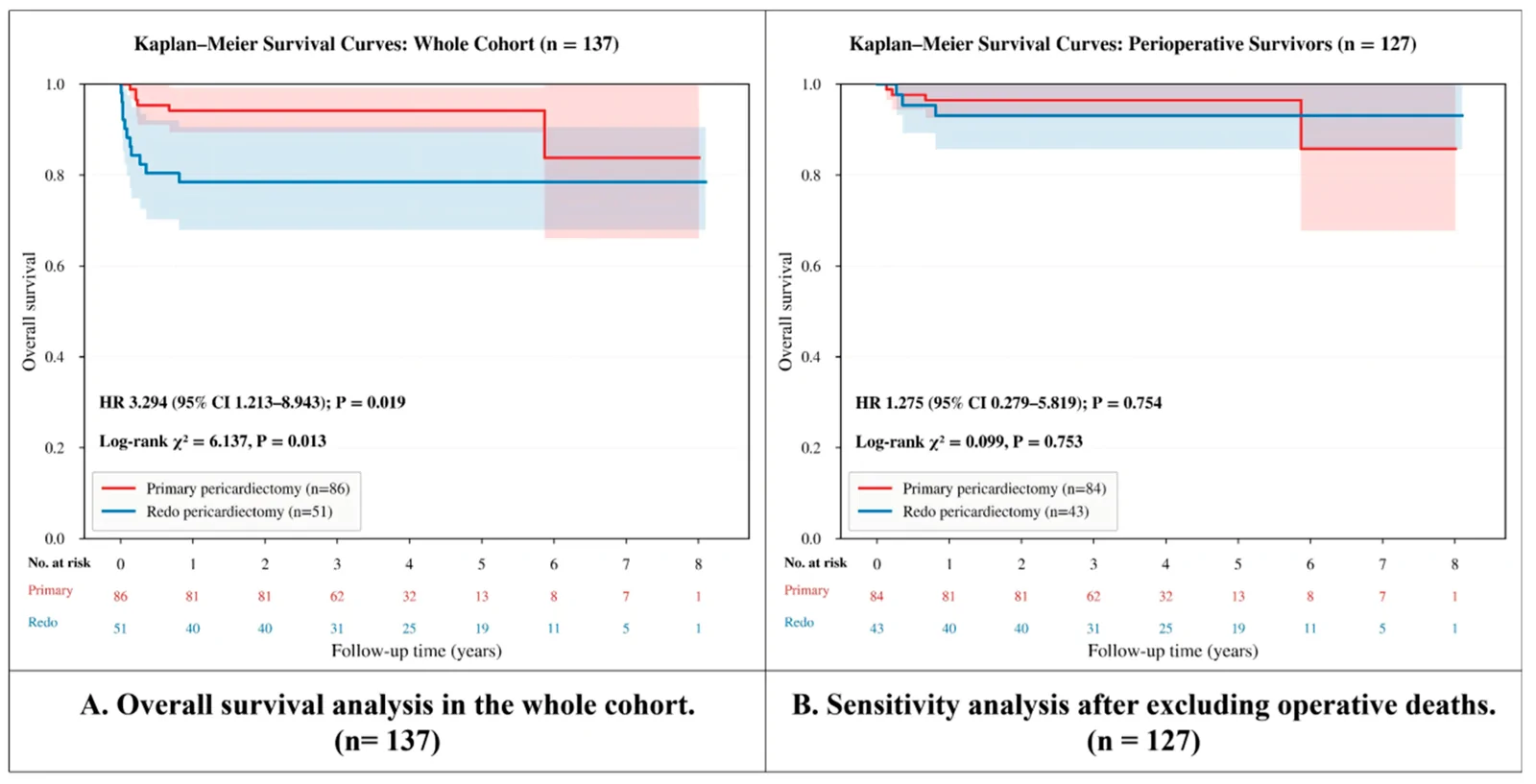
Kaplan–Meier survival curves comparing the primary and redo pericardiectomy cohorts. (**A**) Overall survival analysis in the whole cohort (*n* = 137). The RPC showed significantly lower overall survival than the PPC (HR 3.294, 95% CI 1.213–8.943, *p* = 0.019). (**B**) Landmark analysis from hospital discharge (*n* = 127). After excluding 10 operative deaths (2 in the PPC and 8 in the RPC), no significant difference in long-term survival was observed (HR 1.275, 95% CI 0.279–5.819) between the two cohorts (log-rank *p* = 0.753).

The median follow-up of the entire cohort was 3.6 years (PPC 3.6 vs. RPC 3.7 years, *p* = 0.910). Seventeen patients died of any cause during follow-up: 6 (7.0%) in the PPC and 11 (21.6%) in the RPC. Kaplan–Meier analysis showed significantly lower cumulative overall survival in the RPC (log-rank χ^2^ = 6.137, *p* = 0.013; Table 3 and Figure 2A); univariable Cox regression yielded a hazard ratio (HR) of 3.294 for redo surgery (95% confidence interval [CI] 1.213–8.943, *p* = 0.019).

To account for baseline imbalance, the effect of redo pericardiectomy was additionally adjusted for age, body mass index, and eGFR in a Firth-penalized Cox model. The adjusted hazard ratio remained significant at 2.910 (95% CI 1.001–8.456, *p* = 0.040). Atrial fibrillation and left atrial diameter, which also differed between cohorts, were not included in the adjustment set: both are consequences of long-standing constriction and manifest the disease burden of the redo population, so they lie on the causal pathway between redo surgery and death; including them would constitute over-adjustment and would attenuate the very effect under evaluation.

At prespecified time points (1, 3, and 5 years), Kaplan–Meier estimates of overall survival (95% CI) were 94.2% (89.4–99.3) in the PPC and 78.4% (67.9–90.6) in the RPC, with estimates virtually unchanged across all three time points. Deaths clustered within the first postoperative year (16 of 17), beyond which the two curves ran essentially parallel (Supplementary Tables S3 and S4).

### 3.4. Operative Mortality

Of the 17 deaths from any cause, 10 were operative deaths (2 in the PPC and 8 in the RPC) and 7 occurred after discharge (4 in the PPC and 3 in the RPC; Table 3). Operative mortality was 15.7% in the RPC versus 2.3% in the PPC (relative risk 6.745, 95% CI 1.490–30.542, *p* = 0.006). Because some hospitalizations exceeded 30 days, 30-day mortality alone did not capture all operative deaths: in the RPC, 30-day mortality was 9.8%, with a further 5.9% dying in hospital beyond 30 days, whereas in the PPC, 30-day mortality was 0% and 2.3% died in hospital beyond 30 days.

### 3.5. Secondary Endpoints

The incidence of postoperative LCOS was significantly higher in the RPC than in the PPC (13.7% vs. 3.5%, *p* = 0.026), whereas the rate of unplanned reoperation did not differ between cohorts (*p* = 0.129). The overall incidence of postoperative AKI was also comparable (*p* = 0.354); however, among the 30 patients who developed AKI, the AKI stage was significantly higher in the RPC (*p* = 0.040). Among survivors (*n* = 120), atrial fibrillation remained more prevalent in the RPC (65.0% vs. 40.0%, *p* = 0.010), approximating preoperative levels in both cohorts. Although the distribution of NYHA class among survivors did not differ significantly (*p* = 0.126), the proportion of patients in NYHA class II or higher was 13.8% greater in the RPC (Table 3).

### 3.6. Sensitivity Analysis

Because operative mortality was considerably higher in the RPC, the 10 operative deaths (2 in the PPC and 8 in the RPC) were excluded to examine the influence of early death on mid-term survival. Among patients discharged alive (*n* = 127), long-term survival did not differ between cohorts (log-rank χ^2^ = 0.099, *p* = 0.753; Figure 2B). To examine this more formally, landmark survival analysis beginning at 30 days included 132 patients alive at day 30 (PPC 86, RPC 46), with 6 deaths occurring thereafter in each cohort (HR for redo vs. primary 1.799, 95% CI 0.575–5.627, *p* = 0.306); an analysis beginning at hospital discharge yielded a similarly imprecise estimate (HR 1.275, 95% CI 0.279–5.819, *p* = 0.753; Figure 2B). Thus, among perioperative survivors, no statistically significant difference in long-term survival was detected between cohorts, although confidence intervals were wide and equivalence cannot be inferred from these data.

### 3.7. Special Etiologies

Two uncommon etiologies were represented in this cohort: radiation-induced CP (*n* = 3) and pericardial mesothelioma (*n* = 1). All four patients survived less than 3 months after surgery: the mean postoperative survival of the three patients with radiation-induced CP was 2.0 months, and the patient with mesothelioma survived 2.6 months. These outcomes are descriptive case observations only.

### 3.8. Multivariable Cox Regression for All-Cause Death

Given the small sample and event counts, Firth-penalized Cox regression was used. The final model (apparent C-index 0.907; bootstrap-corrected C-index 0.893; AIC 86.17) included five variables (Table 4). Table 4 reports the pooled estimates across 20 multiple-imputed datasets (Rubin’s rules); missing data were minimal (postoperative albumin 0.7%; preoperative LVEDD 2.9%; all other variables complete) and are detailed by variable and cohort in Supplementary Table S2.

Postoperative LCOS was the strongest independent predictor of death (HR 22.526; 95% CI 5.794–85.102; *p* < 0.001); redo pericardiectomy (HR 3.031; 95% CI 1.034–9.679; *p* = 0.043) and AKI stage (HR 1.848; 95% CI 1.107–3.088; *p* = 0.020) were also independently associated with death. Two exploratory protective associations were noted: a borderline association for postoperative albumin (HR 0.913; *p* = 0.048) and a non-significant trend for preoperative LVEDD (HR 0.920; *p* = 0.089). Missing data and the multiple-imputation procedure are described in detail in Section 2.6.

In a complete-case sensitivity analysis restricted to the 132 patients with no missing values (16 events), the association for redo pericardiectomy was materially unchanged and remained significant (HR 3.514; 95% CI 1.218–11.284; *p* = 0.020), and the associations for LCOS (HR 46.214; *p* < 0.001) and AKI stage (HR 1.664; *p* = 0.049) were preserved in direction and significance. The association for postoperative albumin was directionally similar but no longer statistically significant (HR 0.936; *p* = 0.115), consistent with its borderline nature—the estimate of a weakly significant variable is naturally sensitive to a small change in the analysis set. The protective trend for preoperative LVEDD was essentially unchanged (HR 0.920; *p* = 0.079), in line with its stable association across analytic sets.

With 17 events and the observed allocation of deaths, the study had approximately 80% power to detect a hazard ratio of about 4.1 or larger and only about 60% power to detect the observed hazard ratio of 3.031; the wide confidence intervals around the redo estimate therefore reflect the intrinsic precision limit of this small cohort rather than model instability.

## 4. Discussion

### 4.1. Principal Findings

To our knowledge, this is the largest reported cohort of patients undergoing redo pericardiectomy. Within a single center and time frame, the study provides a direct comparison of primary and redo pericardiectomy and, through a combination of multivariable Cox regression and sensitivity analysis, yields three principal findings. First, redo pericardiectomy was associated with substantially higher perioperative risk, with an operative mortality of 15.7% versus 2.3%. Second, the mid-term survival gap between the cohorts appeared to be concentrated in the perioperative period: in landmark analyses of patients surviving to day 30 (HR 1.799, 95% CI 0.575–5.627) or to hospital discharge (HR 1.275, 95% CI 0.279–5.819), no statistically significant difference in long-term survival was detected, although wide confidence intervals preclude conclusions of equivalence. Third, postoperative LCOS was the strongest independent predictor of death (HR 22.526, *p* < 0.001); each increment in AKI stage increased the hazard of death by 85% (HR 1.848, *p* = 0.020). These predictors highlight potentially modifiable perioperative events as possible targets for improving redo outcomes—hypotheses that remain to be tested. Taken together, these findings suggest that the excess risk associated with redo pericardiectomy is concentrated in the perioperative period rather than persisting into the late period.

### 4.2. Why Is Redo Pericardiectomy Riskier in the Perioperative Period?

The elevated perioperative risk of redo surgery can be understood at anatomic, physiologic, and technical levels. Anatomically, after primary pericardiectomy, the pericardial space is replaced by fibrous scar tissue, and the heart forms dense adhesive planes with the posterior sternal table and mediastinal structures, obliterating normal tissue planes: at re-entry, sternal saw injury to the right ventricle is a material hazard, and during adhesiolysis the coronary arteries, atrial walls, and caval entrances are highly vulnerable [9]. Physiologically, the RPC in this study had a higher prevalence of preoperative atrial fibrillation (64.7% vs. 36.0%) and a larger LAD (54.8 vs. 47.0 mm), reflecting a longer disease course and a heavier constriction burden; long-standing atrial fibrillation abolishes atrial contraction and further weakens the modulation of already impaired diastolic filling by volume loading, laying the groundwork for perioperative low cardiac output. Notably, the RPC was younger (43 vs. 53 years) and had a higher preoperative eGFR (partly reflecting the age difference), yet this favorable profile did not translate into better perioperative outcomes, underscoring the weight of the redo trauma burden. Technically, in the subgroup undergoing pericardiectomy combined with valve or coronary procedures, operative time was significantly longer in the RPC (382.5 vs. 330.0 min, *p* = 0.030), suggesting—exploratorily rather than confirmatorily—a prolonged technical burden associated with redo surgery in complex cases. Moreover, residual diseased pericardium tends to concentrate around the atrioventricular groove, the caval entrances, and the pulmonary veins, precisely the regions where decortication carries the highest risk of myocardial perforation and coronary injury [11].

### 4.3. Prognostic Determinants: A Multidimensional Interpretation

Our multivariable Cox model maps the determinants of postoperative death in CP across five dimensions: cardiac pump function (LCOS), surgical exposure (redo surgery), renal function (AKI stage), nutritional status (postoperative albumin), and structural cardiac compensation (preoperative LVEDD). Several of these findings carry deep pathophysiological implications.

First, postoperative LCOS (HR 22.526) was by far the strongest predictor of death. Its mechanism is rooted in the unique pathophysiology of CP: chronically constrained by a fibrocalcific pericardium, the myocardium undergoes disuse atrophy and loss of compliance; once the constriction is abruptly released, increased venous return floods a low-reserve ventricle that cannot adapt to the sudden change in preload, and the heart lapses into a low-output state [13]. This mechanism was amplified in the RPC: stratified analysis showed that among patients undergoing pericardiectomy combined with valve or coronary procedures, operative time was significantly longer in the RPC (382.5 vs. 330.0 min, *p* = 0.030), whereas no difference was found for isolated pericardiectomy (210.0 vs. 200.0 min, *p* = 0.263). Although formal interaction testing for the subgroup analysis did not reach statistical significance (*p* = 0.551) and the concentration was supported only by stratified subgroup comparisons; this pattern is therefore exploratory. This pattern reveals that the additional trauma burden of redo surgery is not evenly distributed but is concentrated in high-complexity scenarios requiring concomitant procedures, where adhesiolysis and re-entry markedly complicate the establishment of CPB and the handling of multiple structures; operative time is greatly prolonged, and myocardial ischemic time and systemic inflammatory exposure accumulate, forming the direct pathological basis of LCOS [14]. The nearly fourfold LCOS incidence in the RPC (13.7% vs. 3.5%) is the clinical imprint of this mechanistic chain. In summary, the pathophysiological cascade of redo surgery, adhesion and trauma burden (marked by prolonged operative time in concomitant procedures), LCOS, and death provides a mechanistic explanation for the pronounced difference in perioperative mortality between the two cohorts. LCOS may thus represent a modifiable link in this cascade. Whether measures such as prolonged invasive monitoring, protocolized inotropic support, or mechanical circulatory support reduce mortality was not tested in this study; they are presented here as clinical considerations rather than demonstrated implications [15,16].

Second, postoperative AKI showed a clear dose–effect relationship, with each increment in stage increasing the hazard of death by 85%. The high incidence of perioperative renal injury in CP has a dual mechanism: on the one hand, chronic systemic venous congestion raises CVP, impedes renal venous return, and reduces the transmembrane perfusion gradient of the kidney, producing a congestive nephropathy-like state; on the other hand, prerenal hypoperfusion from low cardiac output, high-dose diuretics, and potentially nephrotoxic agents further aggravate tubular injury [17]. This finding implies that perioperative volume management and CVP control matter not only for hemodynamics but also for renal protection.

Third, two protective associations emerged; both should be considered exploratory and hypothesis-generating. The first was a borderline association between higher postoperative albumin and lower mortality: each 1-g/L increment in postoperative albumin was associated with an 8.7% lower hazard of death (*p* = 0.048). In chronic CP, hepatic congestion reduces protein synthesis while intestinal mucosal congestion increases protein loss, so hypoalbuminemia is common. Albumin is also a key determinant of plasma oncotic pressure and an integrated marker of nutritional and inflammatory status, reflecting overall physiologic reserve. Similar protective associations of albumin have been reported in other studies [14,18]. The second was a non-significant protective trend for preoperative LVEDD (HR 0.920, *p* = 0.089). A smaller LVEDD may reflect more severe constriction and a longer disease course: the ventricular myocardium atrophies under long-standing constraint (the so-called small-ventricle state), leaving little ventricular reserve and limited adaptive capacity once the restraint is released. Previous work has likewise linked left ventricular mass to surgical outcomes in CP [14], consistent with the direction of our finding. Both signals arise from a small multivariable model with only 17 events and were not adjusted for multiple testing, and influence diagnostics showed that the albumin coefficient in particular is sensitive to individual observations. These signals should therefore be regarded as hypotheses for testing in larger cohorts, not as established prognostic factors. For LVEDD in particular, adequately powered multicenter studies could clarify this borderline signal by evaluating it together with complementary structural indices such as left ventricular mass index, which integrates chamber size with wall thickness and may better capture the myocardial atrophy underlying chronic constriction.

### 4.4. Special Etiologies: Radiation-Induced and Malignant Disease

Patients with radiation-induced pericarditis (*n* = 3) or pericardial mesothelioma (*n* = 1) in this cohort all survived less than 3 months after surgery. Given the very small size of these subgroups, these observations are descriptive only and cannot support prognostic inference or treatment guidance for these etiologies. In the long-term follow-up study of Bertog and colleagues [19], 7-year survival after pericardiectomy was only 27% in patients with radiation-induced CP, significantly lower than the 88% observed in idiopathic disease, and a history of chest irradiation was an independent risk factor for postoperative death [19,20]. The explanation for this is that thoracic irradiation produces not an isolated pericardial lesion but a pan-cardiac injury simultaneously involving the myocardium, valves, coronary arteries, and mediastinal and pulmonary tissues: constriction and restriction coexist, and although pericardiectomy relieves the mechanical restraint of the pericardium, it cannot reverse the injured myocardium; in addition, these patients frequently carry a history of malignancy and chemotherapy exposure and are in poor general condition, jointly accounting for the dismal prognosis. Primary pericardial mesothelioma is a rare, highly malignant tumor that often presents as pericardial constriction and is difficult to diagnose preoperatively; the reported median survival is only about 6 months, and pericardial resection can only temporarily relieve symptoms without altering the natural history of the tumor [21]. The adverse outcomes of the four patients in our cohort are consistent with these reports.

Beyond the etiologies represented in this cohort, CP can also arise in the setting of rare infiltrative and neoplastic diseases. Erdheim–Chester disease (ECD), a rare non-Langerhans cell histiocytosis with frequent cardiovascular involvement, has been reported to present with constrictive pericarditis together with right atrial infiltration that may require surgical consideration; in a systematic review of published ECD cases, cardiac involvement—including pericardial constriction—emerged as a characteristic feature of the disease phenotype, and its genetic basis (e.g., BRAF mutations) is increasingly recognized [22]. No patient in our cohort had ECD. Nevertheless, these rarer causes reinforce the argument suggested by our radiation-induced and mesothelioma cases that the underlying disease process materially shapes surgical prognosis in CP.

### 4.5. Comparison with Previous Studies

Systematic research on redo pericardiectomy remains scarce and largely confined to small, uncontrolled series. George and colleagues [20] reported 10 redo pericardiectomies with a perioperative mortality of 10.0%, of the same order as the operative mortality in our RPC (15.7%). Ling and colleagues [11] compared left anterolateral thoracotomy with median sternotomy in 24 patients undergoing re-pericardiectomy for recurrent tuberculous constriction, observing a perioperative mortality of 12.5% in both groups; two of the three deaths were attributable to low cardiac output syndrome, reinforcing the central role of LCOS in early redo mortality. In the largest dedicated redo series before ours, Cho and colleagues [9] at the Mayo Clinic analyzed 41 completion pericardiectomies and reported a 30-day mortality of 7% and an in-hospital mortality of 12%, figures closely matching the operative mortality observed in our RPC (15.7%). Notably, they demonstrated that the timing of recurrence critically determines late outcome: patients reoperated within 1 year of the initial procedure achieved a 5-year survival of 73%, whereas those reoperated beyond 1 year had a dismal 5-year survival of only 29% (HR 3.051, *p* = 0.027), prompting the authors to recommend complete pericardiectomy at the initial operation and careful patient selection for late recurrence. In our cohort, the median interval since the initial pericardiectomy was 15 years—far exceeding the 1-year threshold identified by Cho and colleagues—yet long-term survival among perioperative survivors remained favorable (78.4% at 3 years), suggesting that even late redo surgery can achieve acceptable outcomes when perioperative management is optimized.

Two further studies deserve emphasis because they corroborate our core mechanistic findings from independent angles. In a Korean cohort of 90 patients undergoing pericardiectomy, Choi and colleagues [7] identified redo sternotomy as an independent risk factor for long-term mortality (HR 6.441, 95% CI 1.224–33.889, *p* = 0.028), and all four early deaths in their series were caused by low cardiac output syndrome; this observation aligns with our conclusion that redo surgery increases perioperative risk and that LCOS is the dominant driver of early death. Separately, in a retrospective analysis of 3126 patients undergoing right heart catheterization with contrast exposure, Haurand and colleagues [17] demonstrated that venous congestion (right atrial pressure >10 mmHg), rather than low cardiac output per se, was independently associated with acute kidney injury (adjusted HR per 5-mmHg increment in right atrial pressure 1.07, 95% CI 1.01–1.15, *p* = 0.034), whereas cardiac index showed no independent association (*p* = 0.936). This finding refines the interpretation of our own AKI results: although we speculated that both congestion and low output contribute to renal injury, the data of Haurand and colleagues suggest that the congestive component may carry greater weight, underscoring the importance of aggressive perioperative decongestion and CVP control in patients undergoing pericardiectomy. Against this background, the differentiating value of the present study lies in assembling, to our knowledge, the largest redo cohort with a same-center direct comparison and, for the first time, applying a dual strategy of multivariable regression plus sensitivity analysis to precisely localize the source of the survival difference to the perioperative period.

## 5. Clinical Implications

The considerations below are hypotheses derived from observational data; none of the proposed interventions was tested in this study. Our findings have three implications for clinical practice. First, at the level of surgical decision-making, redo surgery should not be withheld from a candidate with a clear indication solely on the assumption that redo operations fare poorly; surgical candidacy must nevertheless be individualized according to etiology, myocardial involvement, comorbidity, technical feasibility, the extent of the previous operation, and patient preference. The focus of decision-making should shift from whether to operate to how to carry the patient safely through the perioperative period. Previous studies have linked radiation-induced CP to poor outcomes [19,20]; for such patients, thorough preoperative assessment of myocardial involvement may be particularly relevant. The present study cannot provide treatment guidance for this etiologic subgroup, which includes only three patients.

Second, at the level of risk stratification, the high-risk features identified in this study (long disease course, atrial fibrillation, marked left atrial enlargement, small LVEDD, and hypoalbuminemia) should be integrated preoperatively to flag patients at high risk of LCOS; hypoalbuminemia should be corrected, volume status optimized to relieve renal venous congestion, and ventricular reserve carefully evaluated.

Third, at the level of perioperative management, preoperative diuresis may be considered in proportion to the degree of edema so that surgery is undertaken close to dry weight; intraoperative volume management should be strengthened, with adequate drainage of pleural and peritoneal effusions to avoid a surge in venous return once the constriction is released, which could precipitate LCOS; and postoperatively the window of invasive hemodynamic monitoring should be extended so that LCOS is recognized and treated early (inotropic agents and, when necessary, mechanical circulatory support), together with a CVP-centered renal protection strategy.

## 6. Limitations

This study has several limitations. First, the single-center retrospective design makes selection and information bias difficult to eliminate entirely. Baseline characteristics were somewhat imbalanced between the two cohorts, particularly age (younger in the RPC) and preoperative atrial fibrillation (more frequent in the RPC); although these differences themselves reflect the clinical phenotype of the redo population, they may confound between-group comparisons. Residual confounding by indication cannot be excluded: the need for redo surgery itself reflects a longer and more severe disease course, and unmeasured factors related to the initial operation may differ between cohorts. In addition, with only 17 events and five covariates, the final model operates near the limit supported by the events-per-variable rule of thumb (approximately 3.4 events per variable). Firth-penalization reduces, but does not eliminate, this constraint. Second, although we assembled the largest redo cohort in the literature (51 patients), the overall sample size (137 patients) and the number of endpoint events (17 deaths) remain limited, constraining the number of covariates that could be entered into the multivariable Cox model and the feasibility of subgroup analyses (for example, stratification by etiology). Third, the median follow-up of about 3.6 years is still insufficient for evaluating truly long-term survival, and extended surveillance will help confirm the late survival trends. Finally, given only 17 events, the multivariable analysis was not adjusted for multiple testing. The associations for postoperative albumin and preoperative LVEDD (borderline and non-significant, respectively, with documented sensitivity to individual observations in influence analyses) should therefore be regarded as exploratory and hypothesis-generating.

Postoperative covariates (LCOS, AKI, postoperative albumin) are by definition unknown at the time of surgery. Our primary model, therefore, provides a postoperative prognostic stratification rather than a preoperative risk estimate. Residual immortal-time and reverse-causation bias cannot be fully excluded in this retrospective design; however, landmark analyses from day 30 and hospital discharge (Section 3.6), which condition on survival to a defined postoperative time point and report effect estimates with confidence intervals, yielded conclusions consistent with the primary analysis. A formal time-dependent Cox model was not feasible because the exact times of onset of LCOS and AKI were not systematically recorded, and such a model with 17 events would be numerically unstable. Because only 17 events occurred, a post-hoc power calculation based on the observed effect would be circular; the precision of the estimates is instead reported directly, and the study had approximately 80% power to detect a hazard ratio of 4.1 or larger.

## 7. Conclusions

In this single-center retrospective cohort study directly comparing primary and redo pericardiectomy in 137 patients with CP, (1) redo pericardiectomy carried significantly higher perioperative risk, with an operative mortality of 15.7% and an LCOS incidence of 13.7%; (2) the survival gap between cohorts was concentrated in the perioperative period; among perioperative survivors, no statistically significant difference in long-term survival was detected (HR 1.275, 95% CI 0.279–5.819), although the estimates were imprecise; and (3) postoperative LCOS was the strongest independent predictor of death, and the excess risk of redo surgery was largely associated with potentially modifiable perioperative events such as LCOS and AKI. These observational findings indicate an association between redo pericardiectomy and higher operative mortality, with estimates that remain imprecise given the small number of events. Surgical candidacy in patients with recurrent or residual constriction should continue to be individualized—taking into account etiology, myocardial involvement, comorbidity, technical feasibility, the extent of the previous operation, and patient preference—and the perioperative strategies discussed above warrant prospective evaluation.

## Figures and Tables

**Table 1:** Baseline characteristics of the study population.

Variable	PPC (*n* = 86)	RPC (*n* = 51)	Statistic (Chi-Square/Z)	*p* Value
Female sex	30 (34.9)	15 (29.4)	0.435	0.510
Age, years	53.0 (40.8–62.0)	43.0 (38.0–56.0)	−2.279	0.023
Admission BMI, kg/m^2^	24.0 (22.3–26.1)	22.9 (20.1–26.2)	−1.993	0.046
Interval since initial pericardiectomy, years	–	15.0 (9.5–29.0)	–	–
**Preoperative NYHA class**				
I	4 (4.7)	2 (3.9)	3.402	0.340
II	20 (23.3)	18 (35.3)		
III	37 (43.0)	22 (43.1)		
IV	25 (29.1)	9 (17.6)		
**Preoperative diuretic weight loss**				
Weight loss, kg	3.6 (2.4–5.7)	4.3 (2.6–6.6)	1.052	0.293
Weight loss, %	5.4 (3.5–8.9)	6.5 (4.1–9.9)	1.489	0.136
**Preoperative rhythm**				
Atrial fibrillation	31 (36.0)	33 (64.7)	10.720	0.002
Sinus rhythm	54 (62.8)	18 (35.3)		
Paced rhythm	1 (1.2)	0 (0.0)		
**Preoperative echocardiography**				
LVEF, %	65.0 (60.0–70.0)	62.0 (58.0–67.5)	−1.237	0.216
LVEDD, mm	40.0 (36.0–45.0)	39.5 (35.0–46.0)	−0.272	0.786
LAD, mm	47.0 (41.5–52.5)	54.8 (46.0–59.0)	3.393	<0.001
**Preoperative laboratory tests**				
Hemoglobin, g/L	134.0 (119.0–145.0)	133.5 (112.5–144.0)	−0.648	0.517
Albumin, g/L	40.3 (37.3–44.3)	39.7 (34.8–43.3)	−1.133	0.257
Creatinine, μmol/L	75.0 (64.0–92.0)	73.5 (62.5–86.0)	−0.877	0.381
Blood urea nitrogen, mmol/L	6.9 (5.3–8.6)	6.7 (4.9–8.3)	−0.606	0.544
Uric acid, μmol/L	447.0 (367.0–583.0)	467.0 (396.5–589.5)	0.793	0.428
eGFR, mL/min/1.73 m^2^	89.1 (71.8–106.0)	99.7 (90.5–114.4)	2.878	0.004

Data are presented as *n* (%) or median (25th and 75th percentiles). BMI, body mass index; NYHA, New York Heart Association; LVEF, left ventricular ejection fraction; LVEDD, left ventricular end-diastolic diameter; LAD, left atrial diameter; eGFR, estimated glomerular filtration rate.

**Table 2:** Perioperative clinical data.

Variable	PPC (*n* = 86)	RPC (*n* = 51)	Statistic (Chi-Square/Z)	*p* Value
**Procedure**				
Isolated pericardiectomy	47 (54.7)	25 (49.0)	0.407	0.523
Concomitant procedure *	39 (45.3)	26 (51.0)		
**ASA class**				
II	5 (5.8)	1 (2.0)	1.092	0.591
III	59 (68.6)	35 (68.6)		
IV	22 (25.6)	15 (29.4)		
**Operative time, min**				
Isolated pericardiectomy subgroup (*n* = 72)	200.0 (162.5–230.0)	210.0 (185.0–240.0)	1.119	0.263
Concomitant procedure subgroup (*n* = 65)	330.0 (300.0–380.0)	382.5 (330.0–445.0)	2.171	0.030
**Cardiopulmonary bypass**				
On-pump (*n* = 66)	40 (46.5)	26 (51.0)	0.256	0.613
Off-pump (*n* = 71)	46 (53.5)	25 (49.0)		
**CPB durations, min (*n* = 66)**				
CPB time	96 (79–122)	147 (131–222)	3.568	<0.001
Aortic cross-clamp time	62 (47–72)	80 (69–102)	3.333	<0.001
Assisted perfusion time	32 (20–38)	39 (27–55)	1.632	0.103
**CVP, cm H_2_O**				
Preoperative	15.0 (10.8–18.0)	12.0 (10.0–16.0)	−1.087	0.277
Postoperative	5.5 (4.0–8.0)	5.0 (4.0–7.0)	−0.758	0.448
Decrease	9.0 (6.0–11.0)	8.0 (6.0–11.0)	−0.970	0.332
*p* value, CVP postoperative vs. preoperative (within group) ^†^	<0.001	<0.001	–	–
**Hospital stay, d**				
Preoperative	7.0 (4.0–10.0)	8.0 (5.0–11.0)	1.633	0.102
ICU stay	2.30 (1.60–3.15)	2.00 (1.58–3.00)	−0.301	0.765
Postoperative	11.00 (7.25–15.00)	10.00 (8.00–12.00)	−1.114	0.265
Total	19.50 (14.25–23.75)	18.00 (15.00–25.00)	0.029	0.977
**Postoperative echocardiography**				
LVEF, %	65.0 (60.0–71.0)	62.0 (60.0–67.0)	−1.888	0.059
LVEDD, mm	41.0 (37.3–45.0)	41.5 (37.0–46.0)	0.693	0.488
*p* value, LVEDD postoperative vs. preoperative (within group) ^†^	0.424	0.183	–	–
LAD, mm	39.0 (35.0–46.0)	46.0 (40.0–57.0)	3.791	<0.001
*p* value, LAD postoperative vs. preoperative (within group) ^†^	<0.001	0.012	–	–
**Etiology**				
Tuberculous	42 (48.8)	29 (56.9)	1.406	0.853
Idiopathic	41 (47.7)	21 (41.2)		
Radiation-induced	2 (2.3)	1 (2.0)		
Mesothelioma	1 (1.2)	0 (0.0)		
**Pre-discharge diuretic weight loss**				
Weight loss, kg	3.50 (2.18–5.58)	4.00 (2.60–6.60)	−1.617	0.106
Weight loss, %	5.20 (3.40–8.86)	6.40 (4.09–9.74)	−1.631	0.103
**Postoperative laboratory tests**				
Hemoglobin, g/L	126.0 (110.0–145.0)	115.5 (100.5–137.5)	−1.812	0.070
Albumin, g/L	39.3 (34.9–43.6)	38.4 (34.8–43.1)	−0.447	0.655
Creatinine, μmol/L	79.0 (65.0–99.0)	73.5 (59.5–96.5)	−0.196	0.844
Blood urea nitrogen, mmol/L	9.9 (7.3–13.1)	9.3 (7.1–12.8)	0.244	0.807
Uric acid, μmol/L	471.0 (344.0–577.0)	421.0 (315.0–563.5)	−0.384	0.701
eGFR, mL/min/1.73 m^2^	85.1 (60.3–104.9)	93.5 (77.4–120.8)	1.357	0.175

Data are presented as *n* (%) or median (25th and 75th percentiles). * Pericardiectomy combined with valve surgery or coronary artery bypass grafting. ^†^ Wilcoxon signed-rank test (within-group comparison). ASA, American Society of Anesthesiologists; CVP, central venous pressure; LVEF, left ventricular ejection fraction; LVEDD, left ventricular end-diastolic diameter; LAD, left atrial diameter; eGFR, estimated glomerular filtration rate.

**Table 3:** Postoperative follow-up outcomes.

Variable	PPC (*n* = 86)	RPC (*n* = 51)	Statistic (Chi-Square/Z)	*p* Value
Follow-up duration, years	3.6 (2.9–4.4)	3.7 (2.5–5.7)	0.114	0.910
**Primary endpoint (*n* = 137)**				
All-cause death ^a^	6 (7.0)	11 (21.6)	6.137 ^†^	0.013
Survival	80 (93.0)	40 (78.4)		
**All-cause death (*n* = 17)**				
Operative death ^a^	2 (2.3)	8 (15.7)	*	0.006
Post-discharge death	4 (4.7)	3 (5.9)		
**Secondary endpoints**				
LCOS	3 (3.5)	7 (13.7)	4.958	0.026
**Unplanned reoperation**				
Reexploration for bleeding	5 (5.8)	1 (2.0)	*	0.129
Redo valve surgery	0 (0.0)	2 (3.9)		
No unplanned reoperation	81 (94.2)	48 (94.1)		
**Postoperative AKI** **(*n* = 30)**	21 (24.4)	9 (17.6)	0.858	0.354
AKI stage 1	17 (19.8)	4 (7.8)	5.611 ^b^	0.040
AKI stage 2	4 (4.7)	3 (5.9)		
AKI stage 3	0 (0.0)	2 (3.9)		
**Atrial fibrillation (survivors, *n* = 120)**				
Yes	32 (40.0)	26 (65.0)	6.674	0.010
No	48 (60.0)	14 (35.0)		
**NYHA class (survivors, *n* = 120)**				
I	67 (83.8)	28 (70.0)	4.970	0.126
II	12 (15.0)	9 (22.5)		
III	1 (1.3)	2 (5.0)		
IV	0 (0.0)	1 (2.5)		

Data are presented as *n* (%) or median (25th, 75th percentiles). ^a^ Operative death as defined in Section 2.4. RPC vs. PPC: RR = 6.745 (95% CI 1.490–30.542). ^b^ AKI stage comparison is restricted to the 30 patients who developed AKI. * Fisher’s exact test. ^†^ All-cause death comparison is the log-rank test of the Kaplan–Meier survival curves; the counts shown are raw event counts. LCOS, low cardiac output syndrome; AKI, acute kidney injury; NYHA, New York Heart Association.

**Table 4:** Multivariable Firth-penalized Cox regression analysis of all-cause death.

Variable	HR	95% CI	*p* Value
Postoperative LCOS	22.526	5.794–85.102	<0.001
Redo pericardiectomy	3.031	1.034–9.679	0.043
Postoperative AKI stage	1.848	1.107–3.088	0.020
Postoperative albumin level	0.913	0.823–0.999	0.048
Preoperative LVEDD	0.920	0.828–1.012	0.089

Estimates are pooled across 20 imputed datasets (*n* = 137) by Rubin’s rules. Model performance: C-index 0.907, bootstrap-corrected 0.893; Akaike information criterion 86.17. HR, hazard ratio; CI, confidence interval; LCOS, low cardiac output syndrome; AKI, acute kidney injury; LVEDD, left ventricular end-diastolic diameter.

## Data Availability

The data underlying this article are available from the corresponding authors upon reasonable request.

## Supplementary Materials

The following supporting information can be downloaded at https://www.mdpi.com/article/10.3390/jcm15187223/s1. Table S1: Candidate covariates evaluated in univariable screening and the final multivariable model; Table S2: Missing data among candidate covariates (*n* = 137); Table S3: Survival estimates at prespecified time points; Table S4: Characteristics and circumstances of the 17 deaths (PPC = primary pericardiectomy cohort; RPC = redo pericardiectomy cohort).

## Abbreviations

AF = atrial fibrillation; AKI = acute kidney injury; ASA = American Society of Anesthesiologists; BMI = body mass index; CABG = coronary artery bypass grafting; CI = confidence interval; CP = constrictive pericarditis; CPB = cardiopulmonary bypass; CVP = central venous pressure; ECMO = extracorporeal membrane oxygenation; eGFR = estimated glomerular filtration rate; HR = hazard ratio; IABP = intra-aortic balloon pump; IQR = interquartile range; KDIGO = kidney disease: Improving Global Outcomes; LAD = left atrial diameter; LCOS = low cardiac output syndrome; LVEDD = left ventricular end-diastolic diameter; LVEF = left ventricular ejection fraction; NYHA = New York Heart Association; PPC = primary pericardiectomy cohort; RPC = redo pericardiectomy cohort; RR = relative risk; TEE = transesophageal echocardiography.
