# Supplementary material for: Perioperative and Mid-Term Outcomes of Primary Versus Redo Pericardiectomy for Constrictive Pericarditis: A Single-Center Retrospective Cohort Study
Source: J Clin Med. 2026 Sep 17;15(18):7223. doi: 10.3390/jcm15187223 (PMC13607922; doi:10.3390/jcm15187223)
Supplement: Supplementary file 1 [file jcm-15-07223-s001.zip › jcm-4529356-supplementary.pdf]

Supplementary

Table S1. Candidate covariates evaluated in univariable screening and the final multivariable model.

Variable	PPC (n=86)	RPC (n=51)	Univariable P	In final model
**Demographic**				
Redo pericardiectomy	86 (100)	51 (100)	0.016	Yes
Female sex	30 (34.9)	15 (29.4)	0.187	No
Age, years	53.0 (41.0–61.8)	43.0 (38.0–55.5)	0.264	No
Admission BMI, kg/m²	24.0 (22.4–26.1)	22.9 (20.1–26.1)	0.076	No
**Preoperative**				
NYHA class (I–IV)	3.0 (2.0–3.0)	2.0 (2.0–3.0)	<0.001	No
Atrial fibrillation	31 (36.0)	33 (64.7)	0.094	No
LVEF, %	65.0 (60.0–70.0)	62.0 (58.0–67.4)	0.413	No
LVEDD, mm	40.0 (36.0–45.0)	39.5 (35.2–45.8)	0.023	Yes
LAD, mm	47.0 (41.5–52.5)	54.8 (46.2–58.8)	0.216	No
Hemoglobin, g/L	134.5 (119.2–146.8)	132.0 (108.0–143.0)	0.165	No
Albumin, g/L	41.0 (37.5–44.5)	39.8 (35.2–43.8)	0.009	No
Creatinine, μmol/L	74.0 (64.0–91.0)	75.5 (64.0–82.0)	0.100	No
Blood urea nitrogen, mmol/L	6.7 (5.3–8.7)	6.6 (4.9–8.3)	0.016	No
Uric acid, μmol/L	441.5 (360.2–574.8)	463.5 (390.0–582.5)	0.012	No
eGFR, mL/min/1.73 m²	90.0 (76.4–105.9)	100.8 (89.3–113.8)	0.115	No
ASA class (II–IV)	3.0 (3.0–3.8)	3.0 (3.0–4.0)	0.077	No
CVP, cm H_2O	15.0 (11.2–18.0)	12.0 (10.5–16.0)	0.107	No
**Intraoperative**				
Concomitant cardiac procedure	39 (45.3)	26 (51.0)	0.286	No
Cardiopulmonary bypass (on-pump)	40 (46.5)	26 (51.0)	0.131	No
Operative time, min	260.0 (190.0–330.0)	295.0 (209.5–382.5)	<0.001	No
**Postoperative**				

CVP, cm H_2O	5.5 (4.2–7.8)	5.0 (4.0–7.0)	0.329	No
Heart rate, bpm	89.5 (81.2–99.0)	90.0 (80.0–97.0)	0.031	No
Hemoglobin, g/L	126.5 (110.8–145.2)	114.0 (100.5–137.5)	0.288	No
Albumin, g/L	39.2 (35.8–43.0)	38.7 (35.6–42.4)	0.005	Yes
Creatinine, μmol/L	78.0 (64.2–97.8)	79.0 (62.2–97.8)	<0.001	No
Blood urea nitrogen, mmol/L	9.6 (7.6–12.7)	10.0 (7.4–14.3)	<0.001	No
Uric acid, μmol/L	458.5 (340.0–575.5)	431.0 (314.0–563.2)	<0.001	No
eGFR, mL/min/1.73 m²	90.0 (63.5–104.9)	92.9 (70.8–119.2)	0.001	No
LVEF, %	65.0 (60.0–71.0)	62.0 (60.0–67.0)	0.088	No
LVEDD, mm	41.0 (37.2–45.0)	41.5 (37.2–46.0)	0.073	No
LAD, mm	39.0 (35.0–46.0)	46.0 (40.2–56.6)	0.099	No
AKI stage (0–3)	0.0 (0.0–0.0)	0.0 (0.0–0.0)	<0.001	Yes
Low cardiac output syndrome	3 (3.5)	7 (13.7)	<0.001	Yes
Unplanned reoperation	5 (5.8)	3 (5.9)	0.009	No

Continuous variables are presented as median (25th–75th percentile); categorical variables as n (%). Univariable P values are from Firth penalized Cox regression for all-cause death. Redo pericardiectomy (the exposure) was retained by design. NYHA class, ASA class and AKI stage were treated as ordinal variables. AKI (binary) was evaluated together with AKI stage but was excluded a priori as redundant with AKI stage. CPB pump/cross-clamp durations and valve-specific details were not included owing to structural non-applicability in off-pump/isolated cases. Etiologic group was not entered as a covariate because two rare subgroups (radiation-induced, n=3; mesothelioma, n=1) were universally fatal and are described separately in Section 3.7.

Table S2. Missing data among candidate covariates (n = 137).

Variable	Overall n (%)	PPC n (%)	RPC n (%)	Imputation
Pre-op LVEF, %	4 (2.9)	3 (3.5)	1 (2.0)	MICE (PMM)
Pre-op LVEDD, mm	4 (2.9)	3 (3.5)	1 (2.0)	MICE (PMM)
Pre-op LAD, mm	4 (2.9)	3 (3.5)	1 (2.0)	MICE (PMM)
Pre-op hemoglobin, g/L	5 (3.6)	4 (4.7)	1 (2.0)	MICE (PMM)
Pre-op albumin, g/L	5 (3.6)	4 (4.7)	1 (2.0)	MICE (PMM)
Pre-op creatinine, μmol/L	6 (4.4)	5 (5.8)	1 (2.0)	MICE (PMM)
Pre-op blood urea nitrogen, mmol/L	6 (4.4)	5 (5.8)	1 (2.0)	MICE (PMM)
Pre-op uric acid, μmol/L	5 (3.6)	4 (4.7)	1 (2.0)	MICE (PMM)
Pre-op eGFR, mL/min/1.73 m^2	6 (4.4)	5 (5.8)	1 (2.0)	MICE (PMM)
Post-op hemoglobin, g/L	1 (0.7)	0 (0.0)	1 (2.0)	MICE (PMM)
Post-op albumin, g/L	1 (0.7)	0 (0.0)	1 (2.0)	MICE (PMM)
Post-op creatinine, μmol/L	1 (0.7)	0 (0.0)	1 (2.0)	MICE (PMM)
Post-op blood urea nitrogen, mmol/L	1 (0.7)	0 (0.0)	1 (2.0)	MICE (PMM)
Post-op uric acid, μmol/L	1 (0.7)	0 (0.0)	1 (2.0)	MICE (PMM)
Post-op eGFR, mL/min/1.73 m^2	1 (0.7)	0 (0.0)	1 (2.0)	MICE (PMM)
Post-op LVEF, %	4 (2.9)	3 (3.5)	1 (2.0)	MICE (PMM)
Post-op LVEDD, mm	4 (2.9)	3 (3.5)	1 (2.0)	MICE (PMM)
Post-op LAD, mm	4 (2.9)	3 (3.5)	1 (2.0)	MICE (PMM)

Percentages use each cohort's denominator (PPC n=86; RPC n=51). All remaining 15 candidate covariates and the outcome variables (follow-up time, vital status) were complete (0% missing). Missing values were assumed missing at random (MAR) conditional on observed covariates and outcomes. Imputation by multivariate imputation with chained equations (m = 20) using predictive mean matching (PMM); the imputation model included all candidate covariates, survival time, and the event indicator.

Table S3. Survival estimates at prespecified time points.

Time point	PPC survival, % (95% CI)	PPC n at risk	RPC survival, % (95% CI)	RPC n at risk
1 year	94.2 (89.4–99.3)	81	78.4 (67.9–90.6)	40
3 years	94.2 (89.4–99.3)	62	78.4 (67.9–90.6)	31
5 years	94.2 (89.4–99.3)	13	78.4 (67.9–90.6)	19

Kaplan–Meier estimates with 95% confidence intervals obtained by log–log transformation. Planned time points were prespecified post hoc for reporting precision as requested by the reviewer; numbers at risk shown at each point. Follow-up was closed on June 15, 2026.

Table S4. Characteristics and circumstances of the 17 deaths (PPC = primary pericardiectomy cohort; RPC = redo pericardiectomy cohort).

No.	Cohort†	Sex	Age, yrs	Pre-op rhythm	Pre-op NYHA	Etiology	Procedure performed	OPT, min	LCOS	Death classification	Post-op survival, months
1	Redo	M	55	AF	III	Tuberculous	pericardiectomy + MVP(30#, CG) + TVP(30#, 3D)	505	Y	In-hospital death	0.1
2	Redo	M	39	AF	II	Tuberculous	pericardiectomy	185	Y	In-hospital death	0.2
3	Redo	F	64	AF	II	Tuberculous	pericardiectomy + TVP(28#, 3D)	290	N	In-hospital death	0.3
4	Redo	M	57	AF	III	Tuberculous	pericardiectomy	170	N	In-hospital death	0.4
5	Redo	M	57	AF	III	Tuberculous	pericardiectomy + MVR(29#, MMV) + TVP(30#, 3D)	950	Y	In-hospital death	0.7
6	Redo	M	51	AF	IV	Idiopathic	pericardiectomy	320	Y	In-hospital death	1.1
7	Redo	F	42	AF	IV	Radiation-induced	pericardiectomy	320	Y	In-hospital death	1.6
8	Primary	M	53	SR	IV	Radiation-induced	pericardiectomy	215	Y	Post-discharge	1.6
9	Redo	M	55	AF	IV	Tuberculous	pericardiectomy + MVP(34#, CG) + TVP(32#, 3D)	670	Y	In-hospital death	1.8
10	Primary	F	71	SR	III	Pericardial mesothelioma	pericardiectomy	200	N	Post-discharge	2.6
11	Primary	M	65	AF	IV	Tuberculous	pericardiectomy + MVR(31#, MMV) + AVR(25#, AMV) + TVP(28#, 3D); *UR: reexploration for bleeding (120 min)*	415	Y	In-hospital death	2.6

12	Primary	M	61	SR	IV	Radiation-induced	pericardiectomy + MVR(27#, MMV) + AVR(23#, AMV)	685	Y	In-hospital death	2.9
13	Redo	M	40	AF	IV	Tuberculous	pericardiectomy; *UR: MVP(30#, CG) + TVP(32#, MC3) (260 min)*	175	Y	Post-discharge death	3.3
14	Redo	M	50	AF	II	Tuberculous	pericardiectomy + MVP(30#, CG) + TVP(30#, MC3)	375	N	Post-discharge	4.3
15	Primary	M	51	SR	III	Tuberculous	pericardiectomy + TVP(30#, MC3) + CABG(LIMA-LAD)	670	N	Post-discharge	8.2
16	Redo	M	43	AF	IV	Tuberculous	pericardiectomy; *UR: MVP(26#, CG) + TVP(27#, BV) (345 min)*	220	N	Post-discharge death	9.9
17	Primary	M	39	SR	IV	Tuberculous	pericardiectomy; *UR: reexploration for bleeding (145 min)*	280	N	Post-discharge death	71.4

Deaths are ordered by postoperative survival time. Postoperative survival is the time from surgery to death (months, one decimal). M, male; F, female; AF, atrial fibrillation; SR, sinus rhythm; NYHA, New York Heart Association; OPT, operative time; LCOS, low cardiac output syndrome; UR, unplanned reoperation; CABG, coronary artery bypass grafting; LIMA, left internal mammary artery; LAD, left anterior descending artery. CG, Medtronic CG Future™ ring; 3D, Medtronic Contour 3D™ ring; MC3, Edwards MC3 ring; BV, Balance Medical Valvuloplasty ring; AMV, aortic mechanical valve (Abbott Regent™); MMV, mitral mechanical valve (Abbott Masters Series). Annuloplasty ring sizes (mm) are shown in parentheses (e.g., 30#). Etiologies were established from the medical history and postoperative pathology reports (the mesothelioma case additionally from pathology). Death classification: in-hospital (n = 10; operative mortality) versus post-discharge (n = 7; during follow-up). No personal identifiers were retained.
